# Baseline Neutrophil-to-Lymphocyte Ratio Is Independently Associated With 90-Day Transplant-Free Mortality in Patients With Cirrhosis

**DOI:** 10.3389/fmed.2021.726950

**Published:** 2021-08-31

**Authors:** Jing Liu, Hai Li, Jie Xia, Xianbo Wang, Yan Huang, Beiling Li, Zhongji Meng, Yanhang Gao, Zhiping Qian, Feng Liu, Xiaobo Lu, Junping Liu, Guohong Deng, Yubao Zheng, Huadong Yan, Liang Qiao, Xiaomei Xiang, Qun Zhang, Ruochan Chen, Jinjun Chen, Sen Luo, La Gao, Liujuan Ji, Jing Li, Xinyi Zhou, Haotang Ren, Sihong Lu, Sumeng Li, Weituo Zhang, Xin Zheng

**Affiliations:** ^1^Department of Infectious Diseases, Institute of Infection and Immunology, Union Hospital, Tongji Medical College, Huazhong University of Science and Technology, Wuhan, China; ^2^Chinese Chronic Liver Failure Consortium, Shanghai, China; ^3^Department of Gastroenterology, Ren Ji Hospital, School of Medicine, Shanghai Jiao Tong University, Shanghai, China; ^4^Department of Infectious Diseases, Southwest Hospital, Third Military Medical University (Army Medical University), Chongqing, China; ^5^Center of Integrative Medicine, Beijing Ditan Hospital, Capital Medical University, Beijing, China; ^6^Department of Infectious Disease, Hunan Key Laboratory of Viral Hepatitis, Xiangya Hospital, Central South University, Hunan, China; ^7^Hepatology Unit, Department of Infectious Diseases, Nanfang Hospital, Southern Medical University, Guangzhou, China; ^8^Department of Infectious Diseases, Hubei Clinical Research Center for Precise Diagnosis and Treatment of Liver Cancer, Taihe Hospital, Hubei University of Medicine, Shiyan, China; ^9^Department of Hepatology, First Hospital of Jilin University, Jilin, China; ^10^Department of Liver Intensive Care Unit, Shanghai Public Health Clinical Centre, Fudan University, Shanghai, China; ^11^Department of Infectious Diseases and Hepatology, The Second Hospital of Shandong University, Shandong, China; ^12^Liver Disease Center, First Affiliated Hospital of Xinjiang Medical University, Xinjiang, China; ^13^Department of Infectious Diseases, Henan Provincial People's Hospital, Henan, China; ^14^Department of Infectious Diseases, The Third Affiliated Hospital, Sun Yat-sen University, Guangzhou, China; ^15^Department of Hepatology, Ningbo No. 2 Hospital, Ningbo, China; ^16^State Key Laboratory for Diagnosis and Treatment of Infectious Diseases, Collaborative Innovation Center for Diagnosis and Treatment of Infectious Disease, The First Affiliated Hospital, Zhejiang University School of Medicine, Hangzhou, China; ^17^Clinical Research Center, Shanghai Jiao Tong University School of Medicine, Shanghai, China

**Keywords:** neutrophil-to-lymphocyte ratio, short-term mortality, cirrhosis, acute decompensation, acute-on-chronic liver failure

## Abstract

**Background:** Patients with cirrhosis have an increased risk of short-term mortality, however, few studies quantify the association between neutrophil-to-lymphocyte ratio (NLR) and 90-day transplant-free mortality in cirrhotic patients.

**Methods:** We prospectively analyzed 3,970 patients with chronic liver diseases from two multicenter cohorts in China (January 2015 to December 2016 and July 2018 to January 2019). Restricted cubic splines (RCS) were used to analyze the relation of NLR and all-causes 90-day transplant-free mortality in cirrhosis.

**Results:** A total of 2,583 cirrhotic patients were enrolled in our study. Restricted cubic splines showed that the odds ratio (OR) of all causes 90-day transplant-free mortality started to increase rapidly until around NLR 6.5, and then was relatively flat (*p* for non-linearity <0.001). The risk of 90-day transplant-free mortality in cirrhotic patients with NLR < 6.5 increased with an increment of 23% for every unit increase in NLR (*p* < 0.001). The patients with NLR < 4.5 had the highest risk (OR: 2.34, 95% CI 1.66–3.28). In multivariable-adjusted stratified analyses, the increase in the incidence of 90-day transplant-free mortality with NLR increasing was consistent (OR >1.0) across all major prespecified subgroups, including infection group (OR: 1.04, 95% CI 1.00–1.09) and non-infection (OR: 1.06, 95% CI 1.02–1.11) group. The trends for NLR and numbers of patients with organ failure varied synchronously and were significantly increased with time from day 7 to day 28.

**Conclusions:** We found a non-linear association between baseline NLR and the adjusted probability of 90-day transplant-free mortality. A certain range of NLR is closely associated with poor short-term prognosis in patients with cirrhosis.

## Introduction

Acute decompensation (AD) is characterized by a rapid deterioration in patients with cirrhosis, leading to the development of acute-on-chronic liver failure (ACLF), a syndrome distinguished by organ failure and high short-term mortality, precipitating events that cause AD in patients with cirrhosis was significantly associated with surrogates of systemic inflammation and increased 90-day mortality (1).

Systemic inflammation plays a crucial role in the pathophysiological pathways leading to the deterioration of disease (2, 3) and was shown to be associated with organ dysfunction and disease progression in patients with AD and ACLF (4–6), inhibition of inflammatory signaling pathway (e.g., LPS-TLR4 axis) ameliorates organ injury and systemic inflammation, preventing disease progression (7). Systemic inflammation was related to alterations in peripheral blood leukocytes that could be captured by simple leucocyte ratio such as neutrophil-to-lymphocyte ratio (NLR) (8). Neutrophil-to-lymphocyte ratio reflects a systemic inflammatory response and shows a significant independent correlation with advanced inflammation diseases (e.g., severe alcoholic hepatitis and non-alcoholic fatty liver diseases) (9, 10). In patients with severe end-stage liver diseases (e.g., ACLF), NLR was identified as an effective biomarker for hospital mortality prediction (11). A previous study reported that increased NLR independently predicted the short-term mortality in cirrhotic patients with AD (12). A nomogram including NLR was used for individual risk stratification and selection of therapeutic strategies (13). The prior systemic review summarized the prognostic ability of NLR in predicting outcomes of cirrhotic patients (14). However, small sample sizes and the heterogeneity of the NLR thresholds used in these studies makes the clinical utility of the NLR somewhat difficult, and the utmost significance of further work should be considered in an attempt to quantify the effect of NLR at a certain range in the clinical setting, and elucidate exact threshold of NLR as well as applicable population.

Based on these considerations, we performed prospective cohorts to analyze the relation of NLR and short-term prognosis, and seek the optimal threshold in which to apply the NLR in stratifying risk, targeting interventions, and assessing resource utilization, contributing to the management of patients with cirrhosis.

## Patients and Methods

### Study Population

Total 3,970 patients with chronic liver diseases were recruited from two prospective multicenter cohorts (NCT02457637, NCT03641872), with 2,600 and 1,370 patients in the development cohorts (from January 2015 to December 2016) and validation cohorts (from July 2018 to January 2019), respectively (15). We collected the patients' demographic characteristics, clinical manifestations, and laboratory measurements from the electronic medical record system. A visualization of the missing values of our data was shown in Supplementary Figure 1. This study was approved by the Ethics Committee of Renji Hospital, Shanghai Jiaotong University School of Medicine, in China, and the ethical committees of each center.

**Figure 1:**
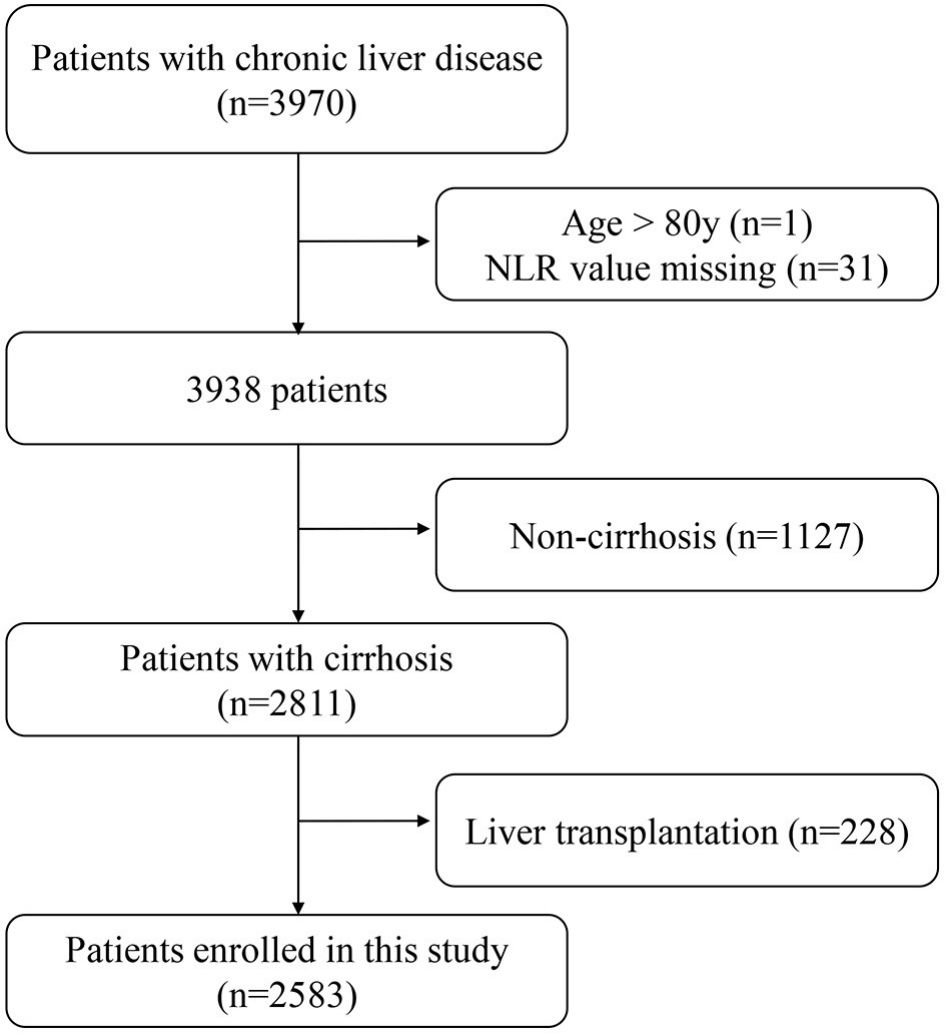
Flow chart of patients enrolled in this study.

The exclusion criteria were as follows: (1) age >80; (2) NLR value missing; (3) non-cirrhosis; (4) liver transplantation. Therefore, the current study was carried out on 2,583 patients (Figure 1).

### Definitions

The diagnosis of liver cirrhosis was confirmed by computed tomography (CT) and magnetic resonance imaging (MRI). Chronic liver disease was defined as liver cirrhosis or a history of liver dysfunction lasting more than 6 months. Acute liver injury was diagnosed by aspartate aminotransferase (AST) or alanine aminotransferase (ALT) >3 × upper limit of normal, or total bilirubin > 2 × upper limits of normal within 1 week before enrollment (15, 16). Acute decompensation was defined by the acute development of hepatic encephalopathy (HE), bacterial infection, overt ascites, gastrointestinal hemorrhage, or any combination of these (Supplementary Table 1), and ACLF was diagnosed according to the European Association for the Study of Liver-Chronic Liver Failure (EASL-CLIF) criteria (17). Definitions related to infections: spontaneous bacteremia, spontaneous bacterial peritonitis, pneumonia, urinary tract infection, and other infections: include cellulitis, acute gastroenteritis, and cholangitis were referred to other studies (18). The MELD score was computed from baseline laboratory parameters based on the standard formula as 3.78 × ln (bilirubin [mg/dl]) + 9.57 × ln (creatinine [mg/dl]) + 11.20 × ln (international normalized ratio) + 6.43 × (0 if cholestatic or alcoholic, 1 otherwise) (19), MELD-Na score was calculated as MELD + (140 – Na [mmol/L]) – 0.025 × MELD × (140 – Na [mmol/L]) (20), and Child-Turcotte-Pugh (CTP) CLIF-C AD, and CLIF-C ACLF scores were calculated as described previously (21–23). Neutrophil-to-lymphocyte ratio was calculated from the ratio of peripheral blood absolute neutrophil and lymphocyte counts. The primary outcome of the study was 90-day transplant-free mortality.

### Statistical Analysis

Categorical variables are expressed as frequencies or percentages and significance was tested by χ^2^ or Fisher's exact test. Parametric quantitative variables are expressed as means ± standard deviation and a *t*-test was used to test the significance. Non-parametric quantitative variables are expressed as median and quartile intervals and the significance was detected by *Kruskal-Wallis* or *Mann-Whitney U*-test. *Kaplan-Meier* analysis was used to assess survival and survival curves were compared using the Log-Rank test.

We also used restricted cubic splines (RCS) with four knots at the 5^th^, 35^th^, 65^th^, and 95^th^ centiles to flexibly model the association of NLR with 90-day transplant-free mortality. In the spline models, age, gender, etiology (HBV, Alcohol, Autoimmune), HE, infection, ascites, gastrointestinal bleeding, total bilirubin (TB), international normalized ratio (INR), and serum creatinine (Scr) were adjusted. We tested for potential non-linearity by using a likelihood ratio test comparing the model with only a linear term against the model with linear and cubic spline terms (24, 25). The diagnostic values of selected parameters were assessed by receiver operating characteristic (ROC) and the area under the ROC curve (AUC).

*P*-value <0.05 was considered statistically significant. SPSS statistical software (Macintosh version 26·0, IBM Corp., Armonk, NY, USA) and R package were used for statistical analysis.

## Results

A total of 2,583 patients with cirrhosis were enrolled in the present study. Based on the NLR quartile, the enrolled patients were divided into four groups, baseline characteristics of the four groups of the patients were listed in Table 1. The average age of the cohort patients was 51.64 ± 11.44 years old and 72.90% of them were male. The main etiology of cirrhosis was chronic infection with HBV (53.93%), followed by alcohol abuse (11.61%), autoimmune-related (9.45%), HBV coupled with alcohol abuse (8.83%), and others. Regarding the complications, ascites were the most common complication with an occurrence rate of 61.87%, while only 692 (26.79%) and 513 (19.86%) patients developed an infection and gastrointestinal bleeding, respectively. Total bilirubin, INR, white blood cells (WBC) levels increased with increasing of NLR, however, there was a significantly decreasing trend in the levels of albumin (ALB), platelet (PLT), hemoglobin, lymphocyte count. For patients with cirrhosis, we found the higher NLR was associated with the higher MELD or MELD-Na scores and 90-day transplant-free mortality.

**Table 1:** Baseline characteristics in all participants.

**Variables**	**Total**	**>4.89**	**(2.77,4.89]**	**(1.74,2.77]**	**(0,1.74]**	***P*** **-value**
	***n*** **= 2,583**	***n*** **= 652**	***n*** **= 638**	***n*** **= 648**	***n*** **= 645**	
Gender (male, %)	1,883 (72.90%)	505 (77.45)	465 (72.88)	472 (72.84)	441 (68.37)	0.004
Age (year)	51.64 (11.44)	52.65 (11.27)	51.52 (11.26)	51.08 (11.73)	51.31 (11.44)	0.065
**Etiology (%)**						
HBV	1,393 (53.93)	331 (50.77)	345 (54.08)	360 (55.56)	357 (55.35)	0.279
Alcohol	300 (11.61)	101 (15.49)	81 (12.70)	59 (9.10)	59 (9.15)	<0.001
Autoimmune	244 (9.45)	44 (6.75)	60 (9.40)	61 (9.41)	79 (12.25)	0.009
HBV and Alcohol	228 (8.83)	63 (9.66)	59 (9.25)	60 (9.26)	46 (7.13)	0.368
HBV and HEV	37 (1.43)	10 (1.53)	8 (1.25)	13 (2.01)	6 (0.93)	0.415
Others	381 (14.75)	102 (15.64)	86 (13.48)	95 (14.66)	98 (15.19)	0.720
**HE grade (%)**						<0.001
1	105 (4.07)	30 (4.60)	23 (3.61)	27 (4.17)	25 (3.88)	
2	118 (4.57)	50 (7.67)	32 (5.02)	20 (3.09)	16 (2.48)	
3	40 (1.55)	21 (3.22)	10 (1.57)	5 (0.77)	4 (0.62)	
4	15 (0.58)	8 (1.23)	6 (0.94)	1 (0.15)	0 (0.00)	
Infection (%)	692 (26.79)	267 (40.95)	181 (28.37)	136 (20.99)	108 (16.74)	<0.001
Ascites (%)	1,598 (61.87)	458 (70.25)	414 (64.89)	371 (57.25)	355 (55.04)	<0.001
GI Bleeding (%)	513 (19.86)	151 (23.16)	145 (22.73)	121 (18.67)	96 (14.88)	<0.001
TB (mg/dl)	3.83 (1.54–13.10)	8.84 (2.06–22.68)	4.95 (1.56–17.92)	3.67 (1.49–11.61)	2.44 (1.34–5.14)	<0.001
INR	1.48 (1.25–1.86)	1.63 (1.39–2.15)	1.48 (1.25–1.88)	1.44 (1.24–1.79)	1.38 (1.20–1.63)	<0.001
CR (mg/dl)	0.78 (0.64–0.97)	0.86 (0.68–1.21)	0.78 (0.64–0.99)	0.75 (0.63–0.90)	0.75 (0.62–0.88)	<0.001
BUN (μmol/L)	5.00 (3.72–7.28)	7.00 (4.60–11.10)	5.10 (3.74–7.31)	4.60 (3.50–6.19)	4.40 (3.40–5.70)	<0.001
ALB (g/L)	30.66 (6.05)	29.70 (5.69)	31.07 (6.34)	31.16 (5.81)	30.70 (6.27)	<0.001
ALT (U/L)	53.00 (26.00–155.00)	48.00 (24.00–144.25)	52.05 (25.77–156.50)	59.60 (28.00–186.40)	50.00 (27.00–131.00)	0.013
AST (U/L)	75.00 (39.00–173.93)	75.00 (34.90–176.30)	78.15 (37.00–179.00)	75.90 (39.20–179.30)	71.00 (43.22–159.07)	0.442
PLT (G/L)	76.00 (50.00–116.00)	77.00 (49.00–117.50)	76.00 (50.00–113.75)	77.00 (52.00–116.25)	75.00 (50.00–116.00)	0.666
AKP (μmol/L)	123.00 (86.00–170.00)	118.00 (78.97–168.00)	120.50 (84.00–170.25)	130.00 (89.00–176.00)	123.00 (90.75–163.00)	0.010
γ-GT (U/L)	64.00 (31.00–126.00)	58.30 (27.00–113.00)	62.00 (31.00–118.40)	70.70 (33.00–147.72)	63.00 (32.75–126.00)	0.004
White blood cell (G/L)	4.74 (3.31–6.92)	7.29 (5.16–10.74)	4.90 (3.50–6.81)	4.21 (2.98–5.90)	3.74 (2.70–4.94)	<0.001
Hemoglobin (g/L)	107.15 (26.81)	104.49 (27.64)	104.57 (27.90)	109.10 (26.77)	110.43 (24.31)	<0.001
Neutrophil count (G/L)	2.99 (1.90–4.85)	5.84 (4.01–9.05)	3.47 (2.42–4.82)	2.58 (1.79–3.63)	1.72 (1.19–2.36)	<0.001
Lymphocyte count (G/L)	1.06 (0.71–1.53)	0.73 (0.49–1.03)	0.96 (0.70–1.30)	1.18 (0.83–1.64)	1.46 (1.09–2.04)	<0.001
K (mmol/L)	3.82 (3.50–4.17)	3.95 (0.75)	3.82 (0.59)	3.81 (0.53)	3.83 (0.53)	<0.001
Na (mmol/L)	137.19 (5.18)	134.95 (6.12)	136.70 (5.12)	138.08 (4.32)	139.04 (3.92)	<0.001
MELD	17.00 (8.25)	21.00 (9.63)	18.00 (8.28)	17.00 (7.19)	14.00 (5.93)	<0.001
MELD-Na	19.00 (10.33)	25.00 (12.51)	20.00 (10.13)	18.00 (8.23)	15.00 (6.93)	<0.001
Child-pugh	9.00 (1.78)	9.00 (1.73)	9.00 (1.75)	9.00 (1.71)	8.00 (1.77)	<0.001
CLIF-C AD	19.00 (3.51)	20.00 (3.46)	19.00 (3.43)	18.00 (3.46)	18.00 (3.41)	<0.001

*HE, hepatic encephalopathy; GI bleeding, gastrointestinal bleeding; TB, total bilirubin; INR, international normalized ratio; CR, serum creatinine; BUN, blood urea nitrogen; ALB, albumin; ALT, alanine aminotransferase; AST, aspartate aminotransferase; PLT, platelet; AKP, alkaline phosphatase; γ-GT, γ-glutamyltransferase; K, blood potassium; Na, serum sodium*.

In univariate analysis, cirrhosis, HE grades, infection, ascites, gastrointestinal bleeding, WBC, TB, INR, BUN, ALT, PLT, hemoglobin, Scr, NLR, Na, K, ALB, MELD, and MELD-Na showed a significant positive correlation with 90-day transplant-free mortality (*p* < 0.05) (data not shown). Multiple regression equations were constructed to further analyze potential relationships between NLR and 90-day transplant-free mortality in different models. Notably, the results revealed that NLR still showed a significant positive relationship with 90-day transplant-free mortality in patients with cirrhosis after adjusting for variables [Model II: age, gender, etiology (HBV, Alcohol, Autoimmune), HE grades, infection, ascites, gastrointestinal bleeding, TB, INR, and Scr (*p* < 0.001)]. To find out the association between subgroup NLR and 90-day transplant-free mortality, we modeled various forms of NLR by multiple regression equation including NLR quartile (≤1.74, >1.74, ≤2.77, >2.77, ≤4.89, >4.89). The results show that the NLR (>4.89) [unadjusted (odds ratio) OR = 7.24, 95% CI 5.05–10.37, *p* < 0.001] and NLR (>2.77, ≤4.89) (unadjusted OR =3.05, 95% CI 2.08–4.46, *p* < 0.001) were related to the incidence of 90-day transplant-free mortality in the univariate analysis compared NLR (≤1.74). It was further confirmed in the multivariate model that the NLR (>4.89) and NLR (>2.77, ≤4.89) were closely associated with 90-day transplant-free mortality adjusted for all variables in Model II (Table 2). When a baseline NLR value of 1.74, 2.77, and 4.89 was used as the cut-off values, patients with NLR >4.89 or NLR >2.77, ≤4.89 groups had a significantly lower 90-day survival probability compared to patients with NLR ≤1.74 or NLR >1.74, ≤2.77 groups (*p* < 0.001) (Supplementary Figure 2).

**Table 2:** Relationship between NLR and 90-day transplant-free mortality.

**Exposure**	**No. of 90-day mortality (%)**	**Model I (Unadjusted)**		**Model II (Fully Adjusted)**	
		**OR (95% CI)**	***P*** **-value**	**OR (95% CI)**	***P*** **-value**
NLR continuous	423 (16.38)	1.14 (1.11, 1.16)	<0.001	1.05 (1.02, 1.08)	<0.001
NLR quartiles					
≤1.74	40 (6.20)	1.0 (Reference)		1.0 (Reference)	
>1.74, ≤2.77	65 (10.03)	1.69 (1.12, 2.54)	0.013	1.14 (0.73, 1.78)	0.551
>2.77, ≤4.89	107 (16.77)	3.05 (2.08, 4.46)	<0.001	1.35 (0.88, 2.08)	0.172
>4.89	211 (32.36)	7.24 (5.05, 10.37)	<0.001	2.46 (1.62, 3.72)	<0.001

*OR, odds ratio; CI, confidence interval; Model I, unadjusted; Model II, adjust age, gender, etiology (HBV, alcohol, autoimmune), hepatic encephalopathy grades, infection, ascites, gastrointestinal bleeding, total bilirubin, international normalized ratio, serum creatinine*.

**Figure 2:**
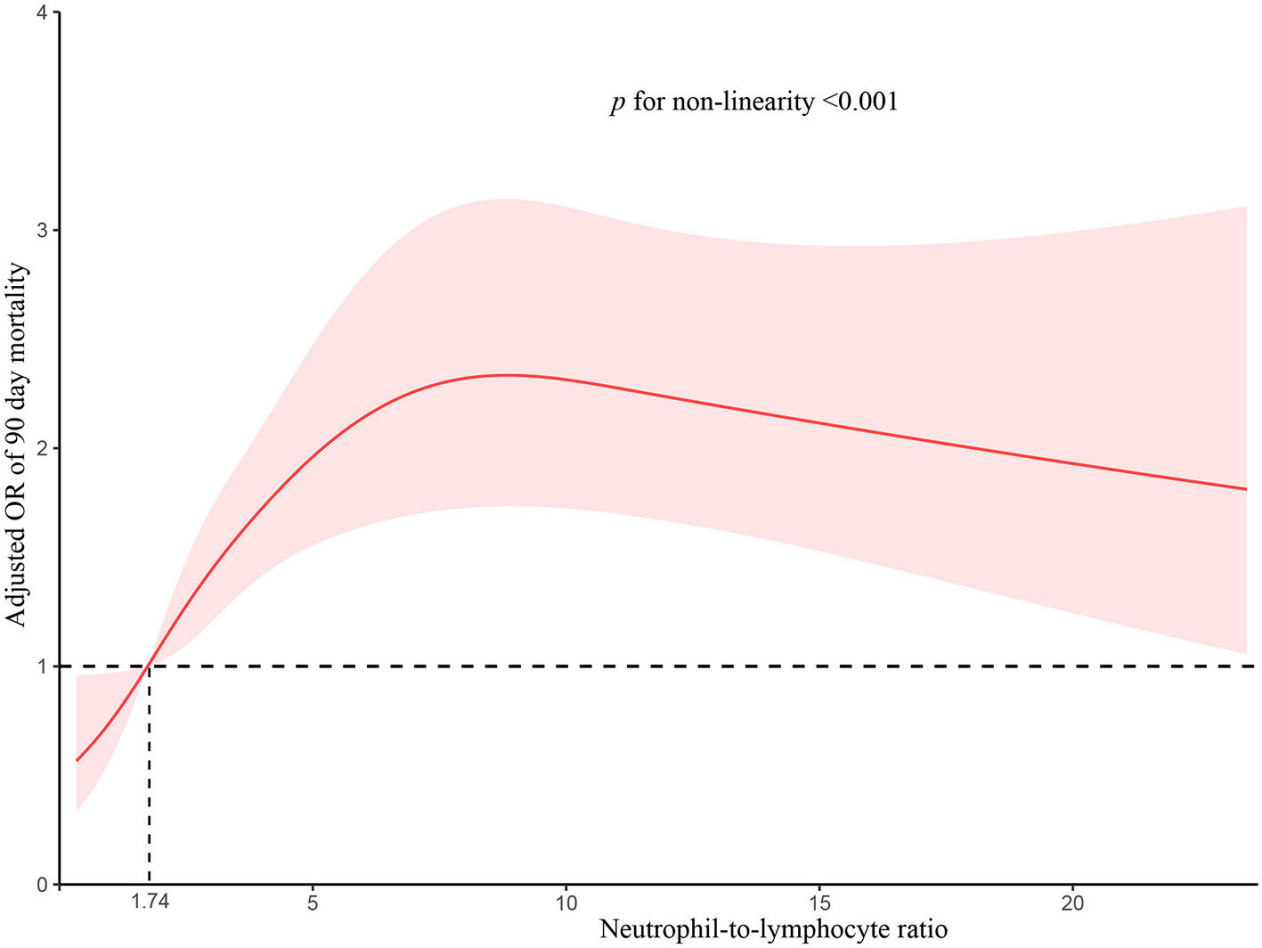
Association of adjusted odds ratio between neutrophil-to-lymphocyte ratio and 90-day transplant-free mortality. The median NLR level within the lowest NLR quartile (1.74) served as the referent value (odds = 1.0). The model was adjusted for age; gender; etiology (HBV, alcohol, autoimmune); cirrhosis; hepatic encephalopathy grades, infection, ascites, gastrointestinal bleeding; total bilirubin; international normalized ratio; serum creatinine. The solid red line represents the multivariable-adjusted odds of mortality as a function of the measured NLR level and 95% confidence intervals are indicated by shaded areas. The *p*-values for non-linearity were <0.001. NLR, neutrophil-to-lymphocyte ratio.

**Figure 3:**
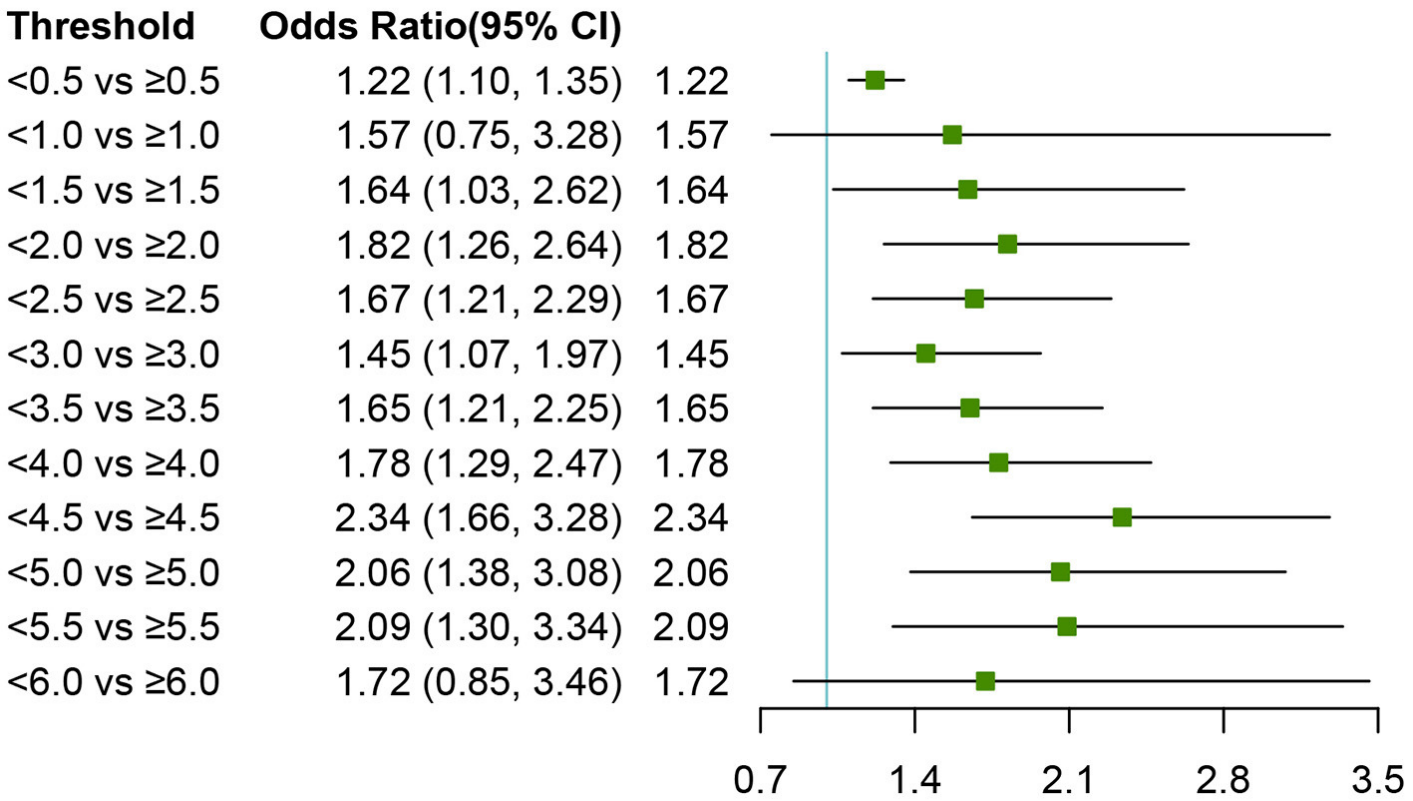
Wide-ranged neutrophil-to-lymphocyte ratio thresholds and adjusted odds ratios of 90-day transplant-free mortality. The cohort of patients (NLR < 6.5) whose NLR was dichotomized into “low” vs. “not low” groups using various cut points in increments of 0.5. Logistic regression was used to estimate the odds ratio of death between the two groups, adjusting for age; gender; etiology (HBV, alcohol, autoimmune); cirrhosis; hepatic encephalopathy grades, infection, ascites, gastrointestinal bleeding; total bilirubin; international normalized ratio; serum creatinine. Squares indicate estimated adjusted odds ratios, and error bars indicate 95% CIs. NLR, neutrophil-to-lymphocyte ratio; OR, Odds Ratios.

We used RCS to flexibly model and visualize the relation of predicted NLR and with all-cause OR of 90-day transplant-free mortality in patients with cirrhosis (Figure 2). The OR-values started to increase rapidly until around 6.5 and then were relatively flat (*p* for non-linearity <0.001). There were 417 cases with NLR >6.5, the fraction of these patients was 16.14% (417/2,583). We next validated the different effects of NLR on OR between the NLR <6.5 and the NLR ≥6.5 groups and found that the threshold-effect in NLR <6.5 group was more obvious than that in NLR ≥6.5 group (Supplementary Figure 3). To assess the exact value-effect of NLR on the 90-day transplant-free mortality, two piece-wise linear regression models were constructed, one for the NLR <6.5 part and the other one for the NLR ≥6.5 part of the trajectory of 90-day transplant-free mortality, as divided by 6.5. Results showed that the risk of 90-day transplant-free mortality in cirrhotic patients with NLR <6.5 increased with an increment of the growth rate of 23% per unit (OR:1.23, 95% CI 1.14–1.32) (*p* < 0.001), similar results were also observed in patients with AD and ACLF (Table 3). To further differentiate low-range NLR populations, we dichotomized the cohort (patients with NLR <6.5) as “low” vs. “not low” using various cut points in increments of 0.5 units. This yields the remarkable result that there is a statistically significant difference in the adjusted probability of 90-day transplant-free mortality for thresholds as low as 1.5 and as high as 5.5, especially for patients with NLR <4.5 (OR:2.34, 95% CI 1.66–3.28) (Figure 3).

**Table 3:** Threshold effect analysis of neutrophil-to-lymphocyte ratio on the 90-day transplant-free mortality using two piece-wise linear regression.

**Neutrophil-to-lymphocyte ratio (NLR)**	**Crude OR (95% Confidence interval) ***P***-value**	Adjusted OR (95% Confidence interval)*P-value
**All patients**		
NLR <6.5	1.47 (1.38, 1.57) <0.001	1.23 (1.14, 1.32) <0.001
NLR ≥ 6.5	0.99 (0.95, 1.03) 0.648	0.98 (0.93, 1.02) 0.321
**AD patients**		
NLR <6.5	1.44 (1.35, 1.54) <0.001	1.23 (1.13, 1.33) <0.001
NLR ≥ 6.5	0.99 (0.96, 1.03) 0.742	0.97 (0.93, 1.02) 0.275
**Non-AD patients**		
NLR <6.5	1.63 (1.33, 1.98) <0.001	1.31 (0.99, 1.73) 0.058
NLR ≥ 6.5	0.93 (0.74, 1.16) 0.497	1.01 (0.78, 1.31) 0.924
**ACLF patients**		
NLR <6.5	1.30 (1.14, 1.49) 0.001	1.23 (1.06, 1.44) 0.007
NLR ≥ 6.5	0.98 (0.92, 1.05) 0.555	0.95 (0.89, 1.02) 0.195
**Non-ACLF patients**		
NLR <6.5	1.36 (1.26, 1.47) <0.001	1.19 (1.08, 1.30) <0.001
NLR ≥ 6.5	0.98 (0.92, 1.03) 0.433	1.00 (0.94, 1.07) 0.940

* *Adjusting for age, gender, etiology (HBV, alcohol, autoimmune), hepatic encephalopathy grades, infection, ascites, gastrointestinal bleeding, total bilirubin, international normalized ratio, serum creatinine*.

**Figure 4:**
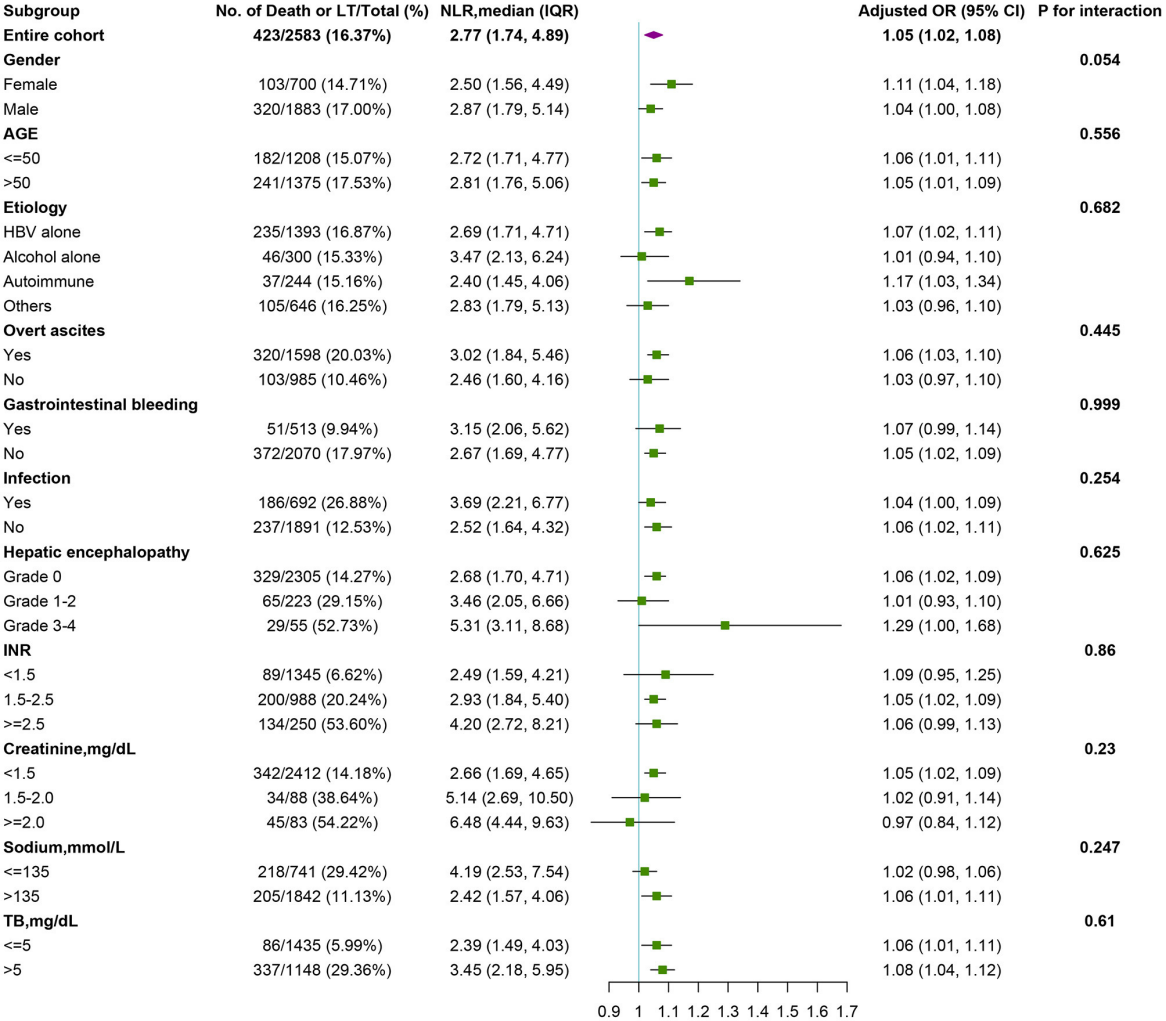
Stratified analyses of risk of death in pre-specified and exploratory subgroups in each subgroup. The multivariable-adjusted odds ratios (95% confidence intervals) of death per unit increment in the standard deviation of NLR level is plotted for the entire cohort and according to strata of baseline covariates. The model was adjusted for age; gender; etiology (HBV, alcohol, autoimmune); cirrhosis; hepatic encephalopathy grades, infection, ascites, gastrointestinal bleeding; total bilirubin; international normalized ratio; serum creatinine. In each case, the model is not adjusted for the stratification variable. A test for interaction between NLR and strata of baseline covariates is also performed. NLR, neutrophil-to-lymphocyte ratio.

Association of the NLR with 90-day transplant-free mortality in patients with cirrhosis was analyzed using logistic regression models across different subgroups, and the results are presented in Figure 4. After adjustment for variables, the increase in the rate of 90-day transplant-free mortality with NLR increasing was consistent (OR >1.0) across all major prespecified subgroups, especially for patients with infection (OR:1.04, 95% CI 1.00–1.09) and non-infection (OR:1.06, 95% CI 1.02–1.11) (Figure 4). Furthermore, we detected no significant interaction for most of the pre-specified baseline factors (*p* > 0.05 for all comparisons).

**Figure 5:**
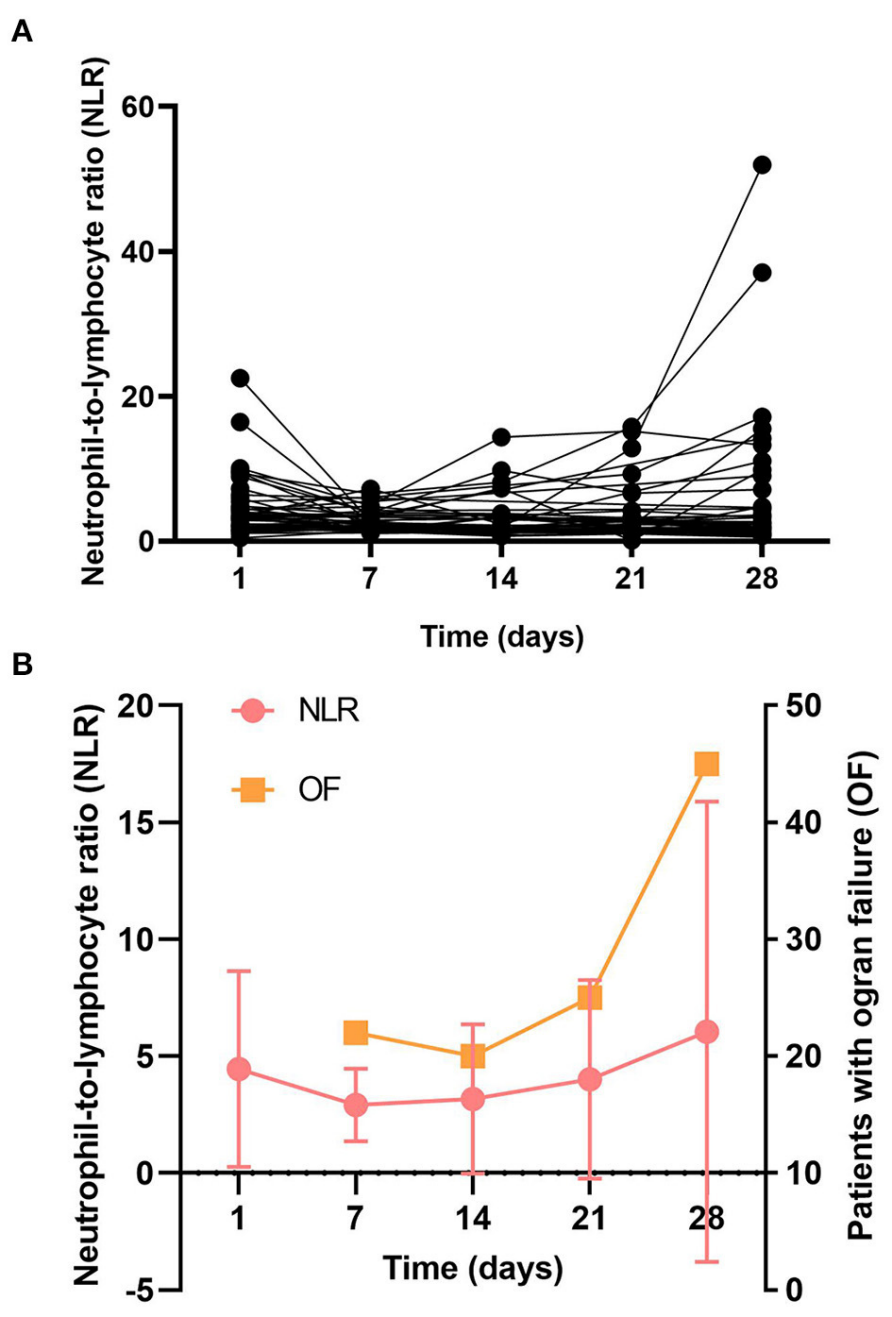
The longitudinal association of NLR and organ dysfunction. Patients were dichotomized into those who did not have organ failure at baseline and subsequently developed organ failure. **(A)** The longitudinal curves of NLR over time; **(B)** Kinetic analysis of the number of organ failure and NLR.

To investigate the longitudinal association between the NLR and organ failure (liver failure, kidney failure, cerebral failure, coagulation failure, circulatory failure, and respiratory failure), we analyzed the kinetic changes of NLR in patients who did not have organ failure at baseline and subsequently developed organ failure during hospitalization, results showed that the trends for NLR and numbers of patients with organ failure varied synchronously, and were significantly increased with time during day 7 to day 28 (Figure 5).

**Figure 6:**
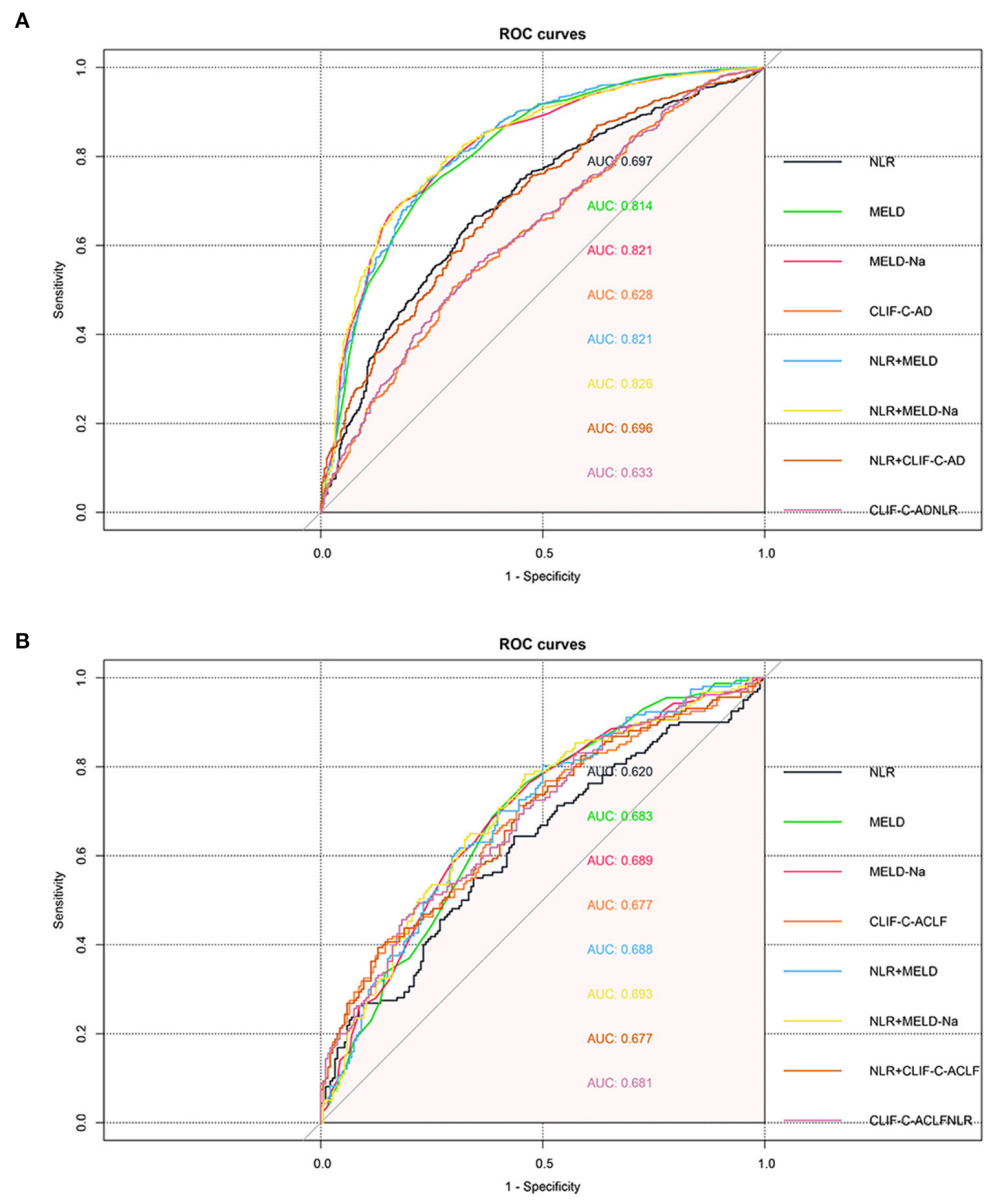
Predictive ability of parameters for 90-day transplant-free mortality. ROC curve and AUC were calculated for neutrophil-to-lymphocyte ratio (NLR), MELD, MELD-Na, CLIF-C AD, CLIF-C ACLF, and and federated parameters by using the R package “pROC.” The cirrhotic patients with AD **(A)** and ACLF **(B)**.

Next, we examined the possibilities of using parameters as prognostic factors for identifying 90-day transplant-free mortality in patients with AD or ACLF. Neutrophil-to-lymphocyte ratio, MELD, MLED-Na, CLIF-C AD, CLIF-C ACLF, and NLR coupled with MELD, MELD-Na, CLIF-C AD, and CLIF-C ACLF were selected as potential prognostic factors for further detailed statistical analysis. The ROC curve and AUC, which were calculated by R package “pROC,” were performed to assess the diagnostic value of these five selected and federated parameters. We used the CLIF-C AD score and CLIF-C ACLF score in patients with AD and ACLF, respectively. Results showed that MELD (0.814) and MELD-Na (0.821) showed better prediction than CLIF-C AD (0.628), and NLR + MELD-Na with a higher AUC (0.826) than NLR + MELD (0.821), MELD-Na (0.821), MELD (0.814) in patients with AD (Figure 6A), however, MELD (0.683), MELD-Na (0.689), and CLIF-C-ACLF (0.677) showed roughly equivalent prognostic power in patients with ACLF (Figure 6B). Additionally, we substituting the WBC in CLIF-C-AD and CLIF-C-ACLF with NLR (CLIF-C ADNLR and CLIF-C ACLFNLR), results showed that it didn't significantly improve score performance [CLIF-C AD vs. CLIF-C ADNLR: 0.628 vs. 0.633 (Figure 6A); CLIF-C ACLF vs. CLIF-C ACLFNLR: 0.677 vs. 0.681 (Figure 6B)].

## Discussion

Our large, multi-center cohort study has firstly confirmed that the value of NLR 6.5 is a feasible cut-off value across clinical settings in patients with cirrhosis, patients with NLR below 6.5 were the optimal population in which to apply NLR in stratifying risk, with a 23% increment in risk of 90-day transplant-free mortality for every unit increase in NLR.

Neutrophil-to-lymphocyte ratio has been studied as a prognostic marker in cirrhotic patients on the basis that it reflects the levels of systemic inflammation driving disease progression in cirrhosis (26, 27). More recently, more and more evidence shows that NLR is a clinically relevant prognostic parameter in patients with liver cirrhosis. A propensity score matching study revealed that NLR over 8.9 may serve as a robust cutoff value for identifying cirrhotic patients at high risk of 90-day mortality (12). Rice et al. (28) revealed that 2.84-folds risk of death was demonstrated in cirrhotic patients with NLR >9 when compared to NLR <3. However, previous studies recruited a unique population, who were predisposed to infection and development of AD or ACLF, representing high mortality, it's unclear if NLR had the same prognostic value of mortality in patients with cirrhosis. In the current study, we found that NLR >4.89 was associated with more than 7-folds risk of 90-day transplant-free mortality compared with the lowest quartile (NLR ≤1.74) in patients with cirrhosis. More importantly, we found that NLR showed a positive relationship with 90-day transplant-free mortality regardless of the presence or absence of infection. These results demonstrated that NLR was an independent risk factor of mortality in hospitalized patients with cirrhosis including AD or non-AD, ACLF or non-ACLF, infection or non-infection patients, suggesting some of the same mechanisms involved in systemic inflammation and eventually resulting in various acute insults. These findings corroborate and extend previous research on the applicable population of NLR.

More recent works on cancer diseases suggested that every unit increase in NLR was associated with a 10–17% increase in 12-month mortality (29, 30). Although NLR has been suggested as an independent risk factor for transplant-free survival and overall survival in liver cirrhosis (11, 26, 28, 31, 32), however, it is still unknown whether there is a linear relationship between the NLR and the risk of death in cirrhosis. Our study provided further results and showed that when an NLR cutoff at approximate 6.5, the increasing trend of OR of 90-day transplant-free mortality with increased NLR occurred significant non-linear relationship or threshold effect, and the risk of 90-day transplant-free mortality in cirrhotic patients with NLR below 6.5 increased with an increment of the growth rate of 23% every unit increase in NLR, especially for patients with AD, ACLF, and non-ACLF. However, the effect was not obvious in NLR over 6.5 group. These results probably indicated that NLR was not enough for comprehensively reflecting the severity of diseases in cirrhotic patients with NLR over 6.5. It's reported that high NLR was not independently associated with overall survival in patients with severe diseases (e.g., advanced nasopharyngeal carcinoma) (33). Therefore, for these patients, we can choose to measure more specific diagnostic markers, such as MELD, a well-known highly specific score that is used to predict short-term survival in patients with a wide spectrum of liver disease (34). Our results also demonstrated that NLR combined with MELD or MELD-Na could improve the effectiveness of predicting mortality.

To our knowledge, MELD scores have been used to predict the mortality of cirrhotic patients awaiting liver transplantation (35), MELD-Na were shown to better predict mortality compared with the MELD score (20, 36), however, predictive significance comparisons among the NLR, MELD, and MELD-Na in cirrhotic patients with large sample size have not been reported before. Therefore, in our study, the original MELD score and the MELD-Na score were included for comparison with other scoring systems. Our results showed that the capacity of MELD-Na and MELD was good in predicting 3-month transplant-free mortality of patients with AD, although the predictive performance of NLR weakened than the other two models, compared MELD-Na and MELD alone, MELD-Na and MELD performed better prediction ability when combined with NLR. However, CLIF-C AD and CLIF-C ACLF didn't show well-predictive performance in our cohort. The heterogeneity of patients enrolled for analysis probably contributes to the difference in predictive accuracy. It's reported that the CLIF-C AD score is more accurate than other liver scores in predicting the prognosis (22) and the CLIF-C ACLF at ACLF diagnosis is superior to the MELD and MELD-Na in predicting mortality (23). However, patients enrolled in these two studies were mainly alcohol-related cirrhosis (49.0 and 54.7%), and the fraction of patients with ascites was high (63.9 and 80.2%), renal insufficiency in these patients were more prone to be observed. In our study, the main etiology of cirrhosis was chronic infection with HBV (53.9%), apart from ascites, 692 (26.79%) and 513 (19.86%) patients developed an infection and gastrointestinal bleeding, respectively. The fraction of patients with liver failure in our cohort was 27.80% (718/2,583), while only 3.2% (83/2,583) patients with renal failure were observed. These results may be indicated that CLIF-C AD or CLIF-C ACLF wasn't the best option for predicting 90-day transplant-free mortality in our cohort.

There are several limitations to our study. First, Although we found that the level of NLR and numbers of patients with organ failure increased synchronously with time during day 7 to day 28, we didn't analyze the correlation between the dynamic change of NLR and short-term mortality, future work needs to focus on providing more powerful evidence. Second, patients with cirrhosis in our multicenter cohorts received varieties of therapies involving different types, doses, and duration of drugs, it's very difficult to homogenize the potential effects of these covariates, thus, we didn't analyze the impacts of antibiotics on the lymphocyte and neutrophil counts, however, stratified analyses showed that infection was not an interaction factor for the impact of NLR on 90-day transplant-free mortality in our cohort, in other words, NLR showed a positive relationship with 90-day transplant-free mortality regardless of the presence or absence of infection, patients with infection are more likely to be treated with antibiotics, these probably indicated NLR was not significantly affected by antibiotics. Furthermore, the NLR threshold of 6.5 has been adjusted for infection.

## Conclusion

Our study concludes that NLR is independently associated with 90-day transplant-free mortality in patients with cirrhosis. Neutrophil-to-lymphocyte ratio >6.5 may serve as a robust cut-off value, and patients with NLR below 6.5 were the optimal population in which to apply the NLR in stratifying risk.

## Data Availability Statement

The original contributions presented in the study are included in the article, further inquiries can be directed to the corresponding author/s at: aclf_group@163.com.

## Ethics Statement

The studies involving human participants were reviewed and approved by the Ethics Committee of Renji Hospital, Shanghai Jiaotong University School of Medicine. The patients/participants provided their written informed consent to participate in this study. Written informed consent was obtained from the individual(s) for the publication of any potentially identifiable images or data included in this article.

## Author Contributions

All authors had access to the data and their role in writing the manuscript. JLiu and XZ: study concept and design. JLiu, HL, JX, XbW, YH, and BlL: contributed equally to this manuscript. HL, GHD, XbW, YH, BlL, ZjM, YHg, ZpQ, FL, XL, JpL, LQ, XmX, QZ, RcC, JjC, SL, LG, LjJ, JLi, XyZ, HtR, ShL, and SmL: data acquisition. WtZ: data analysis. JLiu and XZ: drafted the manuscript. All authors offered critical revision and approved the final draft of the manuscript.

## Conflict of Interest

The authors declare that the research was conducted in the absence of any commercial or financial relationships that could be construed as a potential conflict of interest.

## Publisher's Note

All claims expressed in this article are solely those of the authors and do not necessarily represent those of their affiliated organizations, or those of the publisher, the editors and the reviewers. Any product that may be evaluated in this article, or claim that may be made by its manufacturer, is not guaranteed or endorsed by the publisher.
