# Supplementary material for: Baseline Neutrophil-to-Lymphocyte Ratio Is Independently Associated With 90-Day Transplant-Free Mortality in Patients With Cirrhosis
Source: Front Med (Lausanne). 2021 Aug 31;8:726950. doi: 10.3389/fmed.2021.726950 (PMC8438214; doi:10.3389/fmed.2021.726950)
Supplement: Supplementary file 1 [file Data_Sheet_1.pdf]

Table S1 The definition of acute decompensation of cirrhosis

Acute events	Gold Standard
Acute development of overt ascites	Grade 2 to 3 ascites within less than 2 weeks, according to the International Ascites Club Classification; not included patients with chronic refractory ascites.
Acute hepatic encephalopathy	The acute development of a change in mental status in a patient with previous normal consciousness and no evidence of an acute neurologic disease; not included patients with chronic hepatic encephalopathy.
Acute gastrointestinal hemorrhage	The development of an upper and/or lower gastrointestinal bleeding of any etiology.
Bacterial infections	Spontaneous bacterial peritonitis, spontaneous bacteremia, urinary tract infection, pneumonia, and cellulitis, the most frequent infections in cirrhosis.

Acute decompensation (AD), defined by the acute development of overt ascites, hepatic encephalopathy, gastrointestinal hemorrhage, bacterial infection, or any combination of these.

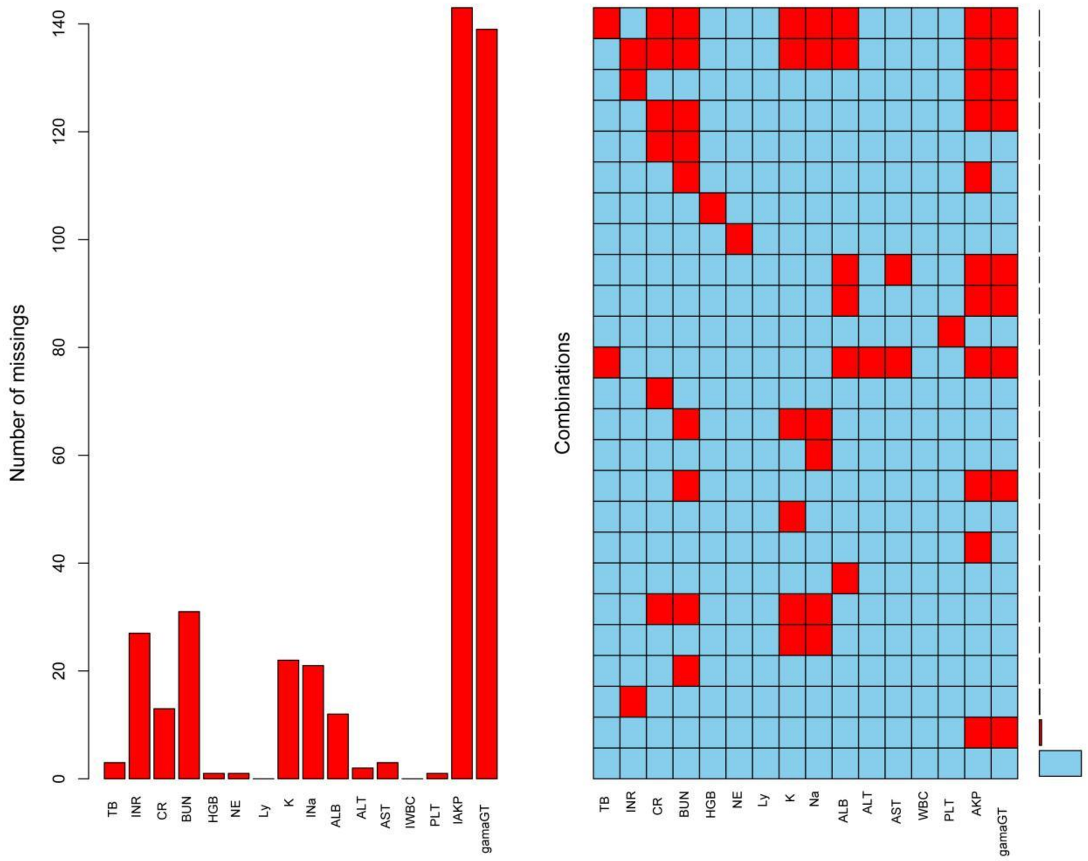

Figure S1. This is a visualization of the missing values of our data. The red bars or squares represent missing data. There are 16 variables in our data, from left to right, the horizontal coordinate stands for TB: total bilirubin, INR: international normalized ratio, CR: serum creatinine, BUN: blood urea nitrogen, HGB: Hemoglobin, NE: neutrophil, Ly: lymphocyte, K: blood potassium, Na: serum sodium, ALB: albumin, ALT: alanine aminotransferase, AST: aspartate aminotransferase, WBC: white blood cells, PLT: platelet, AKP: akaline phosphatase, gama-GT: gama-glutamyltransferase.

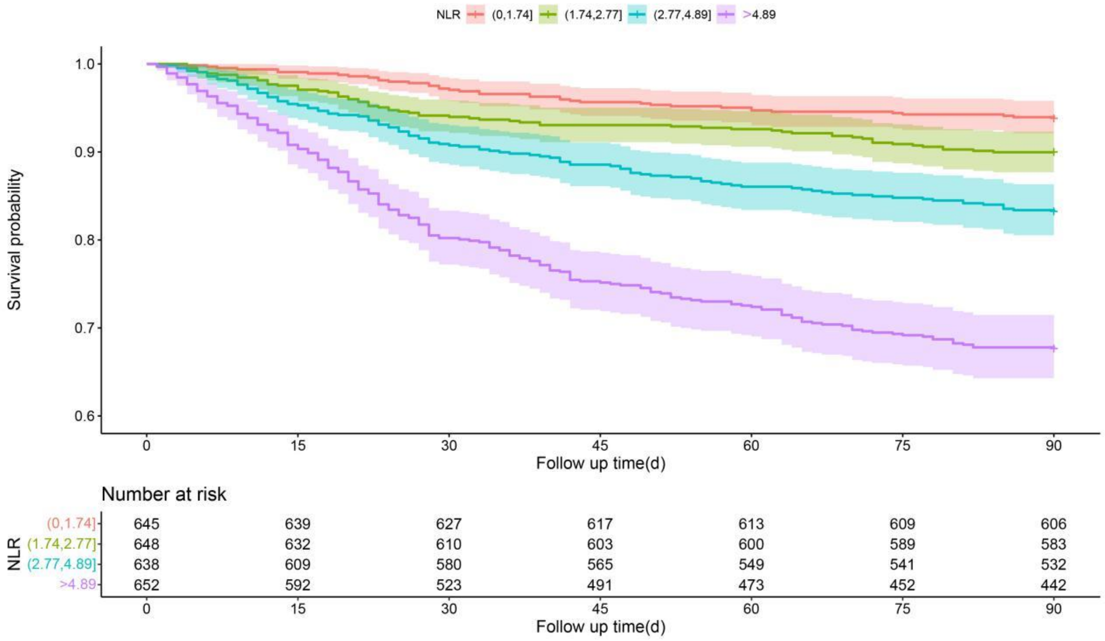

Figure S2. Kaplan-Maier of transplant-free mortality curves in patients with cirrhosis. Patients were stratified according to baseline NLR. Redline: NLR≤1.74; Green line: NLR >1.74, ≤2.77; Cyan-blue line: NLR >2.77, ≤4.89; Purple line: NLR >4.89. 95% confidence intervals are indicated by shaded areas. The p values for the Log-Rank test were less than 0.001. NLR: neutrophil-to-lymphocyte ratio.

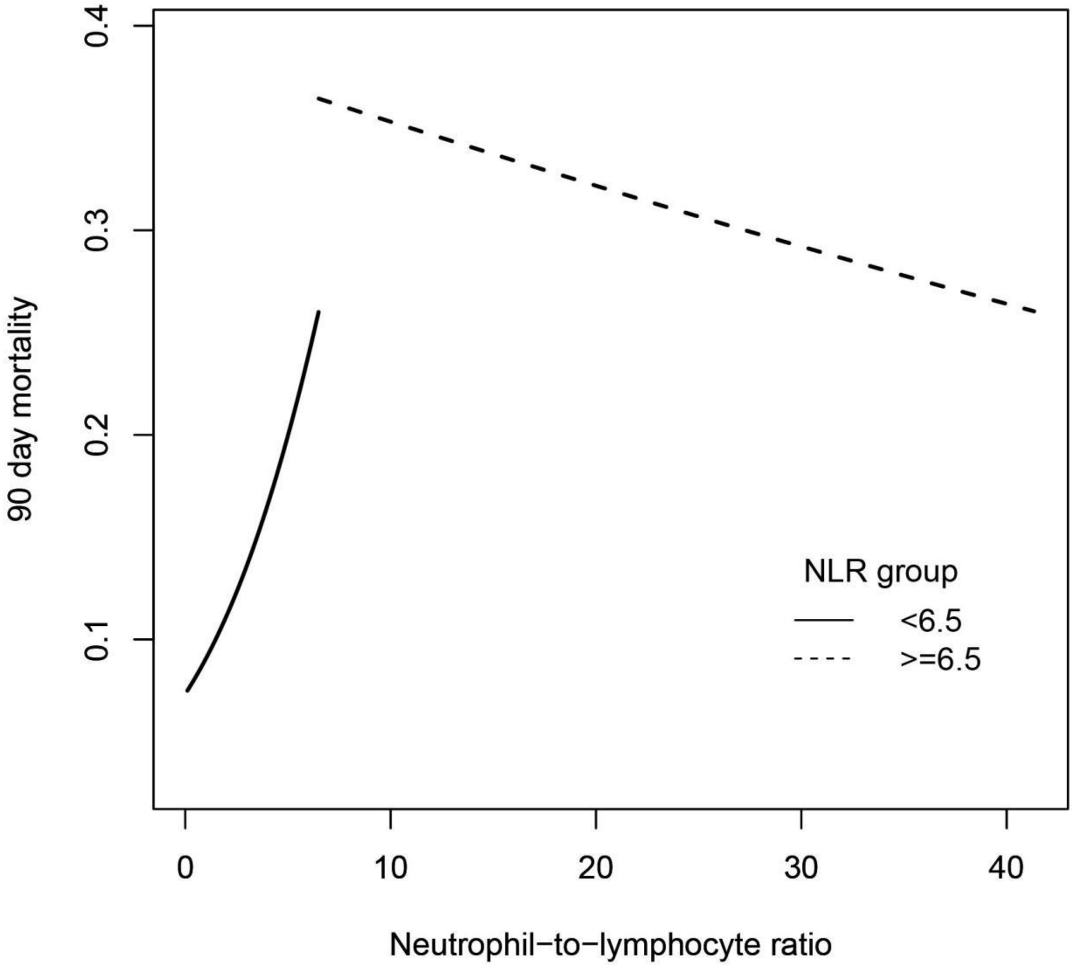

Figure S3. Association of estimate rate between neutrophil-to-lymphocyte ratio (NLR) and 90-day mortality in the cohort of patients (NLR<6.5 and >=6.5).
